# Eco‐Evolutionary Dynamics of Generalist and Specialist Pollinators Facing Plant Diversity Changes

**DOI:** 10.1002/ece3.73182

**Published:** 2026-02-27

**Authors:** Martin Eriksson, Mikael Pontarp

**Affiliations:** ^1^ Centre for Environmental and Climate Science Lund University Lund Sweden; ^2^ Department of Biology Lund University Lund Sweden

**Keywords:** adaptive dynamics, ecosystem services, evolutionary ecology, functional diversity, mutualism, simulations

## Abstract

Changes in plant diversity and abundance due to land‐use modifications can induce plant‐pollinator trophic cascades, potentially leading to long‐term shifts in pollination services. Our ability to mediate such loss of pollination services through informed landscape management is limited by insufficient understanding of long‐term adaptations of wild pollinators to land‐use, especially when accounting for rapid evolution of traits involved in plant‐pollinator interactions. To address this issue, we here use a conceptual trait‐based eco‐evolutionary model to explore how shifts in plant abundance within agricultural landscapes affect: (1) pollinator populations through bottom‐up cascades, and (2) plant populations through top‐down effects of eco‐evolutionary pollinator responses. Our results align with the expectation that specialist pollinators tend to be vulnerable to plant abundance changes over ecological timescales. This vulnerability is exacerbated by limited evolutionary adaptation of specialist pollinators. In contrast, generalists are more resilient to ecological change due to their broader tolerance and, notably, a better capacity for adaptive responses. Such adaptive responses can, however, lead to a significant loss of functional diversity, potentially outweighing the compensatory effects of evolutionary rescue in mitigating negative land‐use change impacts. For specialists, the loss of functional diversity equals the loss of species diversity in our model. By contrast, the loss of functional diversity for pollinators with a more generalised feeding strategy, especially for moderate generalists, may exceed the loss of species diversity to the point that the functional properties of pollinators completely overlap. Our findings demonstrate how resource specialisation influences eco‐evolutionary responses of pollinators to land‐use changes. To ensure stable pollination services, conservation and landscape management strategies must account for limited adaptive capacity of specialists while acknowledging the risk of adaptive loss of functionality in generalists.

## Graphical abstract

We use a trait‐based eco‐evolutionary model to explore how changes in flowering plant composition affect interacting pollinators on short‐term ecological and long‐term evolutionary timescales. We find that evolutionary responses to landscape simplification, especially among generalist pollinators, may lead to a substantial loss of functional diversity within the pollinator community. This loss of functional diversity may outweigh the positive effects of evolutionary rescue in mitigating negative effects of land‐use changes.

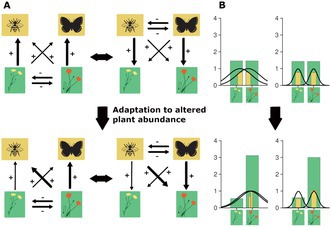

## 1 Introduction

Insufficient abundance or diversity of wild pollinators may have negative effects both on wild flowering plants and on the quality and yield of crops relying on insect pollination (Dicks et al. 2021; Martins et al. 2015; Shi et al. 2024). Therefore, currently declining numbers of pollinators jeopardise both wild biodiversity and agricultural productivity (Potts et al. 2016). There are various reasons for this declining abundance and diversity of pollinators, including the use of pesticides (Roy Chowdhury and Gupta 2025), heavy metal pollution (Musah 2025), and climate change (Raven and Wagner 2021). One of the most important global drivers of declining numbers of pollinators is changes in land cover composition and configuration (Dicks et al. 2021). The mechanisms behind how land‐use influences plant‐pollinator networks are, however, not sufficiently understood. Especially long‐term responses remain elusive (Pontarp et al. 2024).

Land‐use can have both direct and indirect effects on pollinators (Guimarães Jr et al. 2017; Pires et al. 2020). For example, altered plant composition or altered plant functional traits may propagate through bottom‐up cascades from plants to pollinators and further to other species (Zhou et al. 2025). Land‐use‐induced increase or decrease in pollinator abundance or changes in pollinator behaviour or functional traits may also indirectly affect wild flowering plants through top‐down cascades (Hiraiwa and Ushimaru 2017). Since pollinator feeding strategies range from generalists, such as honeybees (Hung et al. 2018), to specialists, such as many solitary bee species (Michez et al. 2008), it is important to consider how specialisation affects how pollinators are influenced by these direct and indirect cascading effects. Indeed, empirical evidence reveals that both plant and pollinator specialists tend to be more sensitive to landscape simplification and habitat fragmentation than generalists (Rader et al. 2014; Weiner et al. 2014; Yan et al. 2022). Therefore, landscape simplification may lead to a landscape dominated by generalist pollinators at the expense of specialists (Gámez‐Virués et al. 2015; Peters et al. 2022). This may have negative consequences for pollination services because generalists may not entirely compensate for the loss of specialised interactions (Maurer et al. 2024). Land‐use effects are, however, difficult to predict, and landscape simplification does not always favour generalists (Dong et al. 2020; Sweeney and Jarzyna 2022).

Moreover, pollinators may evolve new adaptations relatively rapidly (Garnas 2018; McCulloch and Waters 2023). Plant flowering time and the emergence of pollinators have changed during the last century, indicating rapid adaptive responses in phenology (Bartomeus et al. 2011; Weaver and Mallinger 2022). Bumblebee species ( *Bombus balteatus* and *B. sylvicola* ) have been shown to evolve changes in tongue length in response to altered flower morphologies in simplified landscapes (Miller‐Struttmann et al. 2015). Some bees have changed body size in response to habitat fragmentation, which at least in part is likely to be due to evolutionary processes (Oliveira et al. 2016; Warzecha et al. 2016). Such examples of rapid evolution may potentially allow pollinators to adapt to changed environments and thereby rescue them from population decline through evolutionary rescue (Bell 2017; Gomulkiewicz and Holt 1995).

Functional traits play a key role in the interaction between plants and pollinators (Song et al. 2014; Wood et al. 2015). It is therefore useful to focus on functional traits to predict adaptation to changes in plant‐pollinator systems (Opedal 2019, 2021). Together with ecological context and population abundances, functional traits can moreover influence the quality of pollination services (Chase et al. 2023). Functional traits for pollinators can, for example, be the matching between the tongue length of pollinators and the corolla length of plants (Opedal 2021), the matching between activity time of pollinators and flowering time of plants (Weaver and Mallinger 2022), plant traits that attract or reward pollinators (Zariman et al. 2022), or pollinator foraging behaviour (Yourstone 2023). Modelling results by Pontarp et al. (2024) suggest that the functional diversity of pollinators may decrease through evolutionary responses to landscape simplification. Such loss of functional diversity can have a profoundly negative impact on pollination services (Hiraiwa and Ushimaru 2024). However, it has been overlooked how adaptive responses of functional traits differ between generalist and specialist pollinators, although it is expected that they respond very differently to new selection pressures (Anderson et al. 2024; Weiner et al. 2014). Super‐generalists may not need to evolve because they are already sufficiently tolerant, whereas super‐specialists may go extinct due to major change faster than they evolve (Clavel et al. 2011; Reed and Tosh 2019). Case studies also show that adaptive responses to reduced landscape heterogeneity can result in lowered functional diversity of the pollinator community (Pontarp et al. 2024), which may decrease the quality of pollination services (Woodcock et al. 2019). These negative effects may propagate to other parts of the interactive network of wild and domesticated plants and pollinators (Isbell et al. 2011). This may exacerbate declines in pollinator abundance and reduce the long‐term stability and function of the entire ecosystem (Cadotte et al. 2011). Research on whether evolution is positive or negative for the plant‐pollinator community is thus called for (Pontarp et al. 2024). Especially whether evolution is positive or negative in the context of specialist vs. generalist pollinators is an open question.

Empirically disentangling the mechanisms underlying how land‐use influences the composition of plant‐pollinator communities across ecological and evolutionary timescales is challenging due to the inherent complexity of ecological networks (Pontarp et al. 2024). Theoretical models accounting for ecology and evolution of pollinators with varying degrees of specialisation are thus essential to better understand and predict land‐use mediated pollinator declines. Accordingly, we here adopt a trait‐based approach to model the interplay between evolution of pollination‐related functional traits and the population dynamics of plant‐pollinator networks. We consider the matching of a single functional trait of plants and pollinators. Since our model is general, this trait can be taken to represent any relevant trait, or combination of traits, related to pollination. Such traits can, for example, be floral display traits such as size, shape, colours, or scents, reward traits such as nectar volume or nutritional value of nectar or pollen, the behaviour of pollinators with respect to floral display or reward traits, or the physical fit between flowers and pollinators such as corolla and proboscis lengths (Opedal 2019, 2021; Zariman et al. 2022). We investigate how interacting ecological and evolutionary (i.e., eco‐evolutionary) responses depend on how specialised the pollinators are. We explicitly model two mass‐flowering crops and assume that other plants are implicitly included in our model. We use our model to study how changes in the abundance of plant species propagate within a simple plant‐pollinator network on an ecological timescale. We ask: ‘How do plant‐pollinator communities respond to abundance changes of individual plant species on ecological timescales? How does this depend on the specialisation of plant‐pollinator interactions?’ We investigate how functional traits and functional diversity of pollinators evolve as a response to such changes. We ask: ‘What evolutionary responses of pollinators are induced by plant abundance changes, and how do they depend on the specialisation of plant‐pollinator interactions?’ and ‘How does eco‐evolutionary dynamics influence pollinator abundance and diversity, and what are the implications for plant abundance and crop yield?’

## 2 Model

The utility of trait‐based models for studying eco‐evolutionary processes is well established (Georgelin and Loeuille 2016; Pontarp et al. 2019). We design and implement a trait‐based model of a simplified but highly dynamic system of two pollinator and two plant populations, inspired by the model in Pontarp et al. (2024). Hereafter, we refer to these pollinator and plant populations as Pollinator 1, Pollinator 2, Plant 1, and Plant 2. Other plants and pollinators that may potentially be present in the landscape are treated implicitly through their effects on the carrying capacities and growth rates of the plants or pollinators. Similarly, the physical environment is treated implicitly. We assume that functional ecological traits determine the strength of mutualistic interactions between pollinators and plants. These modelled traits can represent virtually any trait of relevance for pollination, for example, morphological flower‐pollinator fit traits, such as the average proboscis length of bees and the average corolla depth of flowers (Opedal 2021), or phenology (Weaver and Mallinger 2022). They can also represent traits such as nectar volume of flowers and foraging behaviour of pollinators, because flowers may experience trade‐offs associated with nectar production and thus have an optimal nectar volume (Pyke and Ren 2023) and pollinators have an optimal foraging strategy that depends on the plant composition in the landscape (Yourstone 2023; Gavini and Quintero 2024). We assume that the strength of the mutualistic benefit is determined by (1) how well the mutualistic traits match, (2) the width of the trait‐matching tolerance between plants and pollinators, and (3) the maximal intrinsic mutualistic benefit for each plant‐pollinator pair. The matching between the functional traits of the pollinators with the functional traits of the plants is an evolving parameter in our model. To account for specialisation, we vary: (1) the width of the trait‐matching tolerance and (2) the relative difference in maximal mutualistic benefits between different plant‐pollinator pairs.

We vary the carrying capacity of one of the two plants (arbitrarily chosen as Plant 1), leading to shifts in plant abundance. These shifts affect pollinator fitness and abundance via trait‐mediated ecological interactions. In response, pollinators may evolve functional traits that enhance their fitness, which feedback to their own and the plant's abundance. We simulate these eco‐evolutionary responses using the adaptive dynamics framework (Geritz et al. 1998) which captures how ecological and evolutionary processes interact. In our model, both plants and pollinators dynamically change their abundance but only pollinators evolve. This reflects the assumption that pollinators are wild and responsive to selection, whereas plants are domesticated and non‐evolving (Loeuille et al. 2013).

### 2.1 Ecological Model

The populations in our trait‐based model grow according to a Lotka‐Volterra‐type of model including competition and mutualism (Murray 2002). Mutualism is maximised when the functional traits of pollinators and plants match (e.g., morphological or phenological matching), and decays with increasing mismatch according to a Gaussian function. We assume that pollinators do not directly compete for floral resources, following common practice in similar models (Häussler et al. 2017; Olsson et al. 2015). This is justified by the abundance of mass‐flowering crops in our modelled study system, which reduces direct resource competition (Bernhardsson et al. 2024). Instead, population regulation is assumed to be driven by other factors like predation or nesting site availability (Sponsler et al. 2023). The plants are assumed to co‐exist in cultivation, with two distinct and non‐evolving trait values, and any competitive advantage for attracting pollinators is assumed to be reflected in how well the plant traits match the pollinators that are present.

More precisely, the interaction‐driven per capita growth of the abundances of plant and pollinator populations is described by the coupled ordinary differential equations (ODEs):

(1) $$\frac{dP_{i}}{P_{i}\mathrm{dt}}=r_{m}\left(1-\sum_{j=1}^{2}\frac{\alpha^{1\left(j\neq i\right)}P_{j}}{K_{i}}\right)+\sum_{j=1}^{2}\theta \varphi^{1\left(j\neq i\right)}\exp\left(-\frac{1}{2}{\left(\frac{\pi_{i}-\rho_{j}}{\sigma }\right)}^{2}\right)R_{j}$$

(2) $$\frac{dR_{i}}{R_{i}\mathrm{dt}}=r_{m}\left(1-\sum_{j=1}^{2}\frac{\alpha^{1\left(j\neq i\right)}R_{j}}{K_{R}}\right)+\sum_{j=1}^{2}\theta \varphi^{1\left(j\neq i\right)}\exp\left(-\frac{1}{2}{\left(\frac{\pi_{j}-\rho_{i}}{\sigma }\right)}^{2}\right)P_{j}$$

where t denotes time; $P_{i}$ and $R_{i}$ are population abundances of plant and pollinator population i=1,2, respectively; $K_{1}$, $K_{2}$, and $K_{R}$ are the carrying capacities for Plant 1, Plant 2, and pollinators, respectively; α=0.5 is the competition coefficient for interspecific competition within the same trophic level; $r_{m}=1$ is the maximal intrinsic growth rate; θ=0.1 and φ are parameters that regulate the strength of mutualism; $\pi_{i}$ and, $\rho_{i}$ are functional trait values of plant and pollinator population i, respectively; and, finally, σ is the width of the trait‐matching tolerance. For simplicity, we assume that σ, which can be considered either as the tolerance of an individual with a given trait or as the standard deviation of the distribution of trait values within the population (or a combination of both), is the same for all pollinator and plant populations. The function $1\left(j\neq i\right)$ is 0 when j=i and 1 otherwise. The parameter θ is the maximal intrinsic mutualistic benefit, whereas φ is the asymmetry in mutualistic benefit. When φ is 0, only a single type of plant can mutualistically benefit a given pollinator, and *vice versa*. By contrast, when φ is 1, the maximal intrinsic mutualistic benefit is equal for both plant‐pollinator pairs.

The model includes two focal plant and pollinator species. One can consider other species to be implicitly treated if their abundances are approximately constant. To see this, assume for example that there are N plant species in total in the landscape, and that the sum of the competition from all the N−2 plants that are not included in the model is $\hat{P}\approx \sum_{i=3}^{N}\alpha_{i}P_{i}$. An expression of the form (1) would be obtained if we rescale the parameters such that $r_{m}={\tilde{r}}_{m}\left(1-\frac{\hat{P}}{K_{1}}\right)$, $K_{1}=\tilde{K}\left(1-\frac{\hat{P}}{\tilde{K}}\right)$, and $r_{m}\theta ={\tilde{r}}_{m}\tilde{\theta }$, where ${\tilde{r}}_{m}$, $\tilde{K}$, and $\tilde{\theta }$ are the true values for maximal intrinsic growth rate, carrying capacity, and maximal intrinsic mutualistic benefit for Plant 1, and similarly for Plant 2 and the pollinators. An analogous argument can be used to implicitly include environmental variation in the model by replacing competition from other species with environmental stochasticity.

We analyse the effect of carrying capacity of Plant 1, $K_{1}$, as a potential effect of fertiliser regimes or farmer decisions of changing the area of land where Plant 1 is grown. We do so for a range of scenarios for φ and σ, which together describe whether the pollinators or plants are generalists or specialists (i.e., key parameters of relevance for the pollinators' responses to environmental change). The remaining constant parameters are chosen to prevent runaway population growth due to mutualism as we analyse different scenarios. To find the ecological equilibrium, an integral part of the eco‐evolutionary analyses (see details below), we solved the system of ODEs given by Equations (1) and (2) with respect to $P_{1}$, $P_{2}$, $R_{1}$, and $R_{2}$, under the condition that all $P_{1}$, $P_{2}$, $R_{1}$, and $R_{2}$ are non‐negative at all time‐points, using the function ode45 in MATLAB (The MathWorks Inc. 2024) Version 24.1.0.2537033 (R2024a).

### 2.2 Eco‐Evolutionary Dynamics

Eco‐evolutionary dynamics is modelled based on classical adaptive dynamics theory (Geritz et al. 1998; Metz et al. 1995), a modelling framework that is still under active development (Buckingham and Ashby 2024; Schmid et al. 2024). This framework assumes that mutations are infrequent and occurring in small steps. That is, the evolving population can be assumed to be at ecological equilibrium when a new mutation occurs, and the mutant phenotype is only slightly different from the resident. The theory also postulates that the number of individuals carrying the mutation is originally negligibly small relative to the abundance of the resident population. This theory is well‐established and has been used to simulate evolution in mutualistic systems, including plant‐pollinator systems (Georgelin and Loeuille 2016; Marcou et al. 2024; Weinbach et al. 2022). Here we use adaptive dynamics to study adaptation of pollination traits ($\rho_{1}$ and $\rho_{2}$) under different scenarios of specialisation (varying parameters σ and φ).

The ecological equilibrium is first found according to the description in the previous subsection (Ecological Model). At this equilibrium, the invasion fitness of a mutant of Pollinator 1 or Pollinator 2, denoted here as $f_{1}$ or $f_{2}$, is

(3) $$\begin{array}{c} f_{1}\left(\rho^{\prime },P_{1}^{*},P_{2}^{*},R_{1}^{*},R_{2}^{*}\right)=1-\frac{R_{1}^{*}+0.5R_{2}^{*}}{K_{R}}\\ +0.1\left[\exp\left(-\frac{{\left(\pi_{1}-\rho^{\prime }\right)}^{2}}{2\sigma^{2}}\right)P_{1}^{*}+\varphi \exp\left(-\frac{{\left(\pi_{2}-\rho^{\prime }\right)}^{2}}{2\sigma^{2}}\right)P_{2}^{*}\right] \end{array}$$

(4) $$\begin{array}{c} f_{2}\left(\rho^{\prime },P_{1}^{*},P_{2}^{*},R_{1}^{*},R_{2}^{*}\right)=1-\frac{{0.5R_{1}^{*}+R}_{2}^{*}}{K_{R}}\\ +0.1\left[\varphi \exp\left(-\frac{{\left(\pi_{1}-\rho^{\prime }\right)}^{2}}{2\sigma^{2}}\right)P_{1}^{*}+\exp\left(-\frac{{\left(\pi_{2}-\rho^{\prime }\right)}^{2}}{2\sigma^{2}}\right)P_{2}^{*}\right] \end{array}$$

where $P_{1}^{*}$, $P_{2}^{*}$, $R_{1}^{*}$, and $R_{2}^{*}$ denote equilibrium population densities and $\rho^{\prime }$ denotes the mutant trait value. Note that the invasion fitness of the mutant does not directly depend on the resident trait value. This is a direct consequence of our choice of modelling mass‐flowering crops, making competition for flowers negligible and simplifying the ecological model as well as the evolutionary analysis. Selection on ρ is driven exclusively by population abundances, thus allowing us to focus on landscape effects and the proportion of plants within. The selection gradients, $s_{1}$ and $s_{2}$, for Pollinator 1 and Pollinator 2 are mathematically formulated as the derivatives of Equations (3) and (4) with respect to $\rho^{\prime }$ evaluated at $\rho_{1}$ and $\rho_{2}$. That is

(5) $$\begin{array}{c} s_{1}={\left.\frac{df_{1}\left(\;\rho^{\prime },\rho ,P_{1}^{*},P_{2}^{*},R_{1}^{*},R_{2}^{*}\right)}{{\mathrm{d\rho}}^{\prime }}\right|}_{\rho \prime =\rho_{1}}\\ =\theta \frac{\pi_{1}-\rho_{1}}{\sigma^{2}}e^{-\frac{{\left(\pi_{1}-\rho_{1}\right)}^{2}}{2\sigma^{2}}}P_{1}^{*}+\theta \varphi \frac{\pi_{2}-\rho_{1}}{\sigma^{2}}e^{-\frac{{\left(\pi_{2}-\rho_{1}\right)}^{2}}{2\sigma^{2}}}P_{2}^{*} \end{array}$$

(6) $$\begin{array}{cc} s_{2} & ={\left.\frac{df_{2}\left(\;\rho^{\prime },\rho ,P_{1}^{*},P_{2}^{*},R_{1}^{*},R_{2}^{*}\right)}{{\mathrm{d\rho}}^{\prime }}\right|}_{\rho \prime =\rho_{2}}\\ & =\theta \varphi \frac{\pi_{1}-\rho_{2}}{\sigma^{2}}e^{-\frac{{\left(\pi_{1}-\rho_{2}\right)}^{2}}{2\sigma^{2}}}P_{1}^{*}+\theta \frac{\pi_{2}-\rho_{2}}{\sigma^{2}}e^{-\frac{{\left(\pi_{2}-\rho_{2}\right)}^{2}}{2\sigma^{2}}}P_{2}^{*} \end{array}$$

A population phenotype adapts in the direction of positive fitness gradient, that is, towards an increasing growth rate. The phenotype change after each such favourable mutation is proportional to the speed of evolution according to the canonical equation (Dieckmann and Law 1996; Geritz et al. 1998).

A key aim of our study is to identify the evolutionarily stable state (ESS) where no mutant types have positive invasion fitness and evolution thus stops. The ESS is analysed for different simulated land‐use scenarios as follows. As the landscape composition changes, a new equilibrium state for population abundances is attained and pollinator traits experience novel selection pressures. After a new favourable mutation has been established, a new ecological equilibrium is computed for a resident population with this new phenotype according to Equations ((1), (2)). The process is repeated until $\max\left(s_{1},s_{2}\right)<10^{-6}$. Once both selection gradients are below this critical point, the system is assumed to have reached an ESS. Alternatively, the algorithm is terminated once the population abundance of any pollinator population is sufficiently close to zero (i.e., smaller than $10^{-3}$) at the same time as the selection gradient for the other pollinator population is below the critical value. In this case, the pollinator with the low abundance is considered to have gone extinct. Similarly, the algorithm is terminated once the population abundance of any plant gets smaller than $10^{-3}$ at the same time as one of the pollinators is close to an ESS adapted to the other plant.

### 2.3 Analysis of Results and Examples of Outcomes

We initiate the model with equal plant abundances by setting $K_{1}=K_{2}=2$, making the initial plant carrying capacities twice as large as the carrying capacities of pollinators, which are scaled to 1. The functional trait values of the plants are fixed at $\pi_{1}=1$ and $\pi_{2}=3$ making plant‐pollinator interactions specialised for σ≤1. From these conditions, pollinator traits are allowed to evolve until the system reaches its initial ESS, which defines the baseline abundances of plants and pollinators. To explore how plant carrying capacity influences eco‐evolutionary dynamics, we vary $K_{1}$ across a range of 8 values of σ and 3 values of φ, in total 24 combinations of σ and φ (Table 1). For each combination, we examine: (1) the ecological equilibrium abundances of pollinators and plants after the change in carrying capacity, and (2) the pollinator trait values at the new ESS after allowing pollinator traits to evolve under the altered conditions. We thus, measure abundance of both plants and pollinators, but because we assume that only pollinators evolve (as the plants are assumed to be domesticated and thus not under natural selection), we only measure the trait values for pollinators (Table 2). These population abundances and trait values are used to quantify the pollinator community diversity at the new ESS using two different diversity metrics. To calculate the species richness, we use the Gini–Simpson index (Simpson 1949), which for our two‐pollinator system is

(7) $$2\left(1-\lambda \right)=2\left(1-\frac{{\left(R_{1}^{*}\right)}^{2}+{\left(R_{2}^{*}\right)}^{2}}{{\left(R_{1}^{*}+R_{2}^{*}\right)}^{2}}\right)$$

**TABLE 1:** List of parameters and their notation.

Parameter	Explanation	Values
σ	Width of trait matching tolerance	0.25, 0.5, 0.75, 1.0, 1.25, 1.5, 1.75, 2.0
θ	Maximal intrinsic mutualistic benefit	0.1
φ	Asymmetry in mutualistic benefit	0.25, 0.5, 0.75
$K_{1}$	Carrying capacity of Plant 1	1–4
$K_{2}$	Carrying capacity of Plant 2	2
$K_{P}$	Carrying capacity of pollinators	1
$\pi_{1}$	Trait value of Plant 1	1
$\pi_{2}$	Trait value of Plant 2	3
α	Interspecific competition	0.5
$r_{m}$	Maximal intrinsic growth rate	1

**TABLE 2:** List of variables and their notation. Equilibrium abundances refer to ecological equilibrium.

Variable	Explanation
$P_{1}$	Population abundance of Plant 1
$P_{2}$	Population abundance of Plant 2
$R_{1}$	Population abundance of Pollinator 1
$R_{2}$	Population abundance of Pollinator 2
$P_{1}^{*}$	Equilibrium abundance of Plant 1
$P_{2}^{*}$	Equilibrium abundance of Plant 2
$R_{1}^{*}$	Equilibrium abundance of Pollinator 1
$R_{2}^{*}$	Equilibrium abundance of Pollinator 2
$\rho_{1}$	Trait value of Pollinator 1
$\rho_{2}$	Trait value of Pollinator 2

The Gini‐Simpson index is multiplied by 2 to scale it to a number between 0 and 1. To calculate functional diversity, we use Rao's quadratic entropy (Rao 1982), which for our two‐pollinator system is calculated as follows:

(8) $$Q=2\frac{R_{1}^{*}R_{2}^{*}}{{\left(R_{1}^{*}+R_{2}^{*}\right)}^{2}}|\rho_{1}-\rho_{2}|.$$

## 3 Results

**FIGURE 1:**
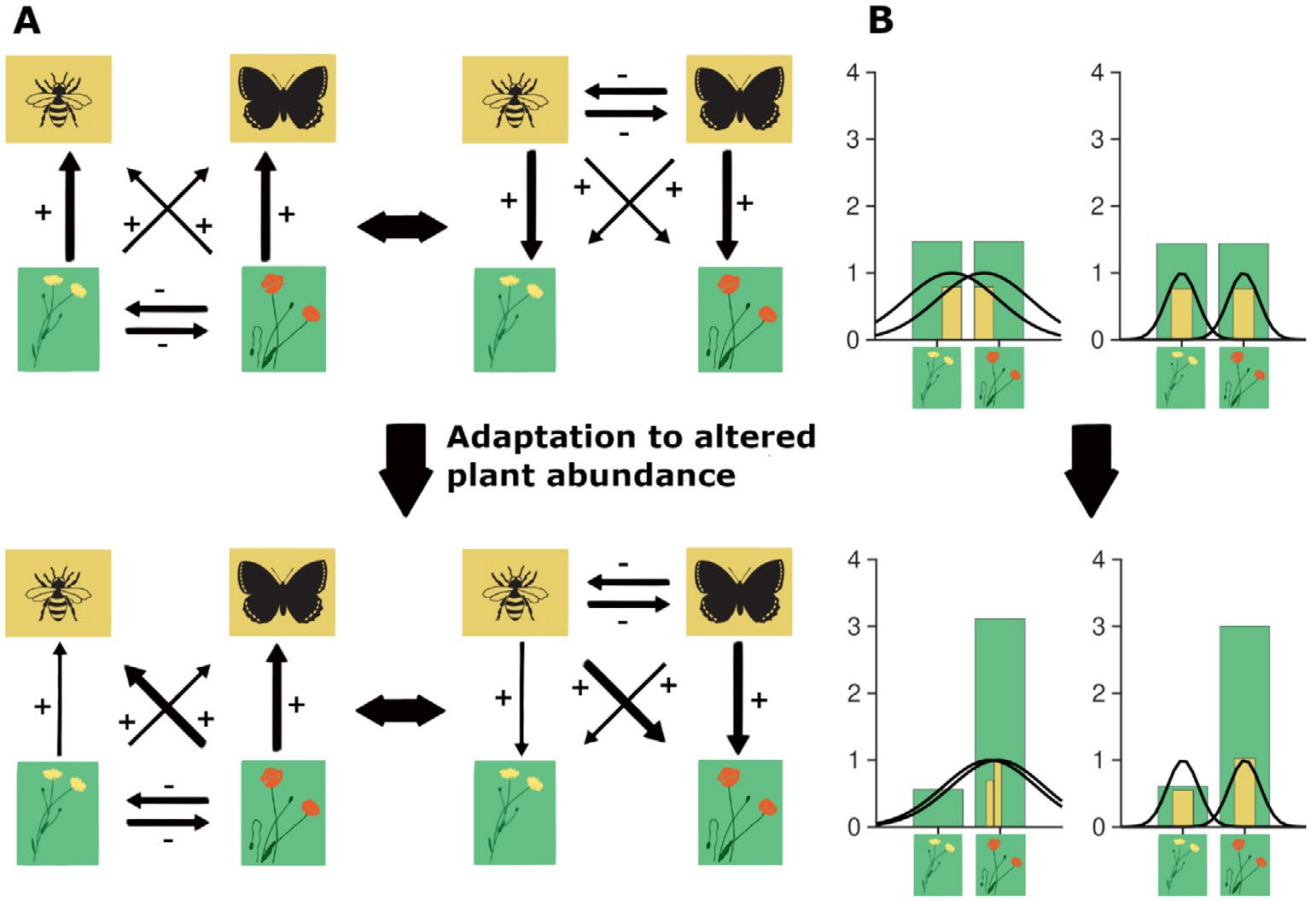
Illustration of the model with competition, and mutualistic interactions (A), and the difference between evolutionary responses of generalists and specialists (B). Plants interact with other plants through competition, and with pollinators through mutualistic bottom‐up effects (left‐hand side of panel A). Pollinators interact with other pollinators through competition, and with plants through mutualistic top‐down effects (right‐hand side of panel A). For both plants and pollinators, the mutualistic interactions are stronger when the functional traits of plants and pollinators match (illustrated with thicker arrows). Adaptation to altered plant composition drive evolution of the functional traits of pollinators which can alter the interaction network such that both pollinators match better with the most abundant plant (lower row). Panel (B) illustrates that the evolutionary equilibrium differs between moderate generalists and specialists. The centre of each bar indicates the optimal trait value for plants (green) and pollinators (yellow). The height of each bar indicates the abundance of the corresponding population. Solid black curves indicate the relative mutualistic benefit for plants provided by pollinators as a function of the plant's trait value. Whereas moderately generalist pollinators strongly adapt to the more abundant plant, specialists do not undergo any noticeable evolution. Conceptual results are illustrated for σ=1.5 (generalists, left‐hand side) and σ=0.5 (specialists, right‐hand side). In the bottom row of panel B, the bars that indicate pollinator abundances for the generalists are made narrower to facilitate distinction between separate populations. Figure adapted from artwork by Anna Sofie Persson.

Figure 1 schematically illustrates the model and highlights how the width of the trait matching tolerance (σ) influence how pollinators respond ecologically and evolutionarily to changes in plant carrying capacity. On an ecological timescale, plants and pollinators interact through a feedback loop between trait‐dependent mutualistic bottom‐up and top‐down effects (Figure 1A). Note that the bottom‐up and top‐down effects are driven both by abundances and by trait values of the pollinators and plants. Increased abundance of a plant leads to the following chain of effects (illustrated in the upper row in Figure 1A): (1) both competition with the other plant and the mutualistic bottom‐up effects with pollinators increase, (2) this influences the abundance of pollinators, and pollinators whose functional traits match the plant trait well increases in abundance, (3) increased abundance of a pollinator increases both competition with the other pollinators and the mutualistic top‐down effects on plants leading to further abundance changes of plants and pollinators, (4) a feedback loop between these processes proceeds until an ecological equilibrium is reached. At this ecological equilibrium, pollinators experience selection for better matching between their functional traits and the traits of the dominant plant (illustrated in the lower row of Figure 1A): (1) adaptive changes in trait value increase top‐down and bottom‐up effects for the more abundant plant (the red flower in our illustration) and decrease them for the less abundant plant, (2) this perturbs the network from the equilibrium and a induces a new feedback loop between bottom‐up and top‐down effects until a new ecological equilibrium is reached, (3) the process continues until the selection gradient diminishes. In this case, an evolutionarily stable state (ESS) has been reached.

How much the evolutionary responses alter the trait values of the pollinators, however, differs considerably between specialists and moderate generalists (Figure 1B). While moderate generalists may adapt fully to the dominant plant, specialists exhibit negligible evolution. This is because specialists are unable to utilise an alternative plant, no matter how abundant it is, whereas generalists may switch to utilising a plant that they match relatively poorly with if it is sufficiently abundant, which will induce directional selection towards this plant. Below we expand on these general results in the context of short ecological timescale responses and long‐term adaptive responses to modelled land‐use change.

### 3.1 Effects of Plant Abundance Changes on Short Ecological Timescales

The pollinator abundances at ecological equilibrium ($R_{1}^{*}$ and $R_{2}^{*}$) are proportional to the carrying capacity, $K_{1}$, of Plant 1 (Figure S1). The proportionality is positive for $R_{1}^{*}$ and negative for $R_{2}^{*}$. The slope ($R_{i}^{*}/K_{1}$) of this linear relationship between $R_{i}^{*}$ and $K_{1}$ depends on σ and φ. Pollinators with smaller σ are more strongly positively or negatively affected by changes in plant composition than pollinators with larger σ (Table 3 and Figure S1). There is almost no difference between σ=0.25 and σ=0.5 and no effect of φ when σ≤0.5. This is because σ≤0.5 is too small relative to the distance between the mean trait values of the two plants (i.e., $\left|\pi_{1}-\pi_{2}\right|=2$) for pollinators to utilise their non‐preferred plant for foraging. For larger σ, larger values of φ make pollinators considerably less affected by changes in plant composition compared to when φ is small (Table 3). Thus, for small σ and/or φ, land‐use changes can induce considerable decreases in species diversity on ecological timescales.

**TABLE 3:** Sensitivity of equilibrium pollinator abundances to plant abundance change.

σ	φ=0.25		φ=0.5		φ=0.75	
	A	B	A	B	A	B
0.25	0.256	−0.205	0.256	−0.205	0.256	−0.205
0.5	0.256	−0.205	0.256	−0.205	0.256	−0.205
0.75	0.254	−0.203	0.252	−0.201	0.250	−0.198
1	0.246	−0.194	0.231	−0.176	0.201	−0.144
1.25	0.235	−0.181	0.199	−0.140	0.135	−0.070
1.5	0.226	−0.170	0.179	−0.116	0.115	−0.043
1.75	0.220	−0.162	0.169	−0.103	0.109	−0.032
2	0.216	−0.157	0.164	−0.095	0.107	−0.027

*Note:* The numbers in this table indicate how much the equilibrium abundance of pollinators changes when $K_{1}$ changes (i.e., $R_{i}^{*}/K_{1}$), for $R_{i}^{*}=R_{1}^{*}$ (A) and $R_{i}^{*}=R_{2}^{*}$ (B). Larger numbers in absolute value means that the pollinator populations are more sensitive to changes in $K_{1}$. Positive numbers indicate a positive correlation whereas negative numbers indicate a negative correlation. The parameter φ denotes the asymmetry in mutualistic benefit between different plant‐pollinator pairs (with 0 indicating that only one of the plants gives a mutualistic benefit, whereas 1 means that the maximal mutualistic benefit is equal for both plants) and σ denotes the width of the trait matching tolerance of plants and pollinators.

### 3.2 Selective Pressures Induced by Changes in Plant Abundance

**FIGURE 2:**
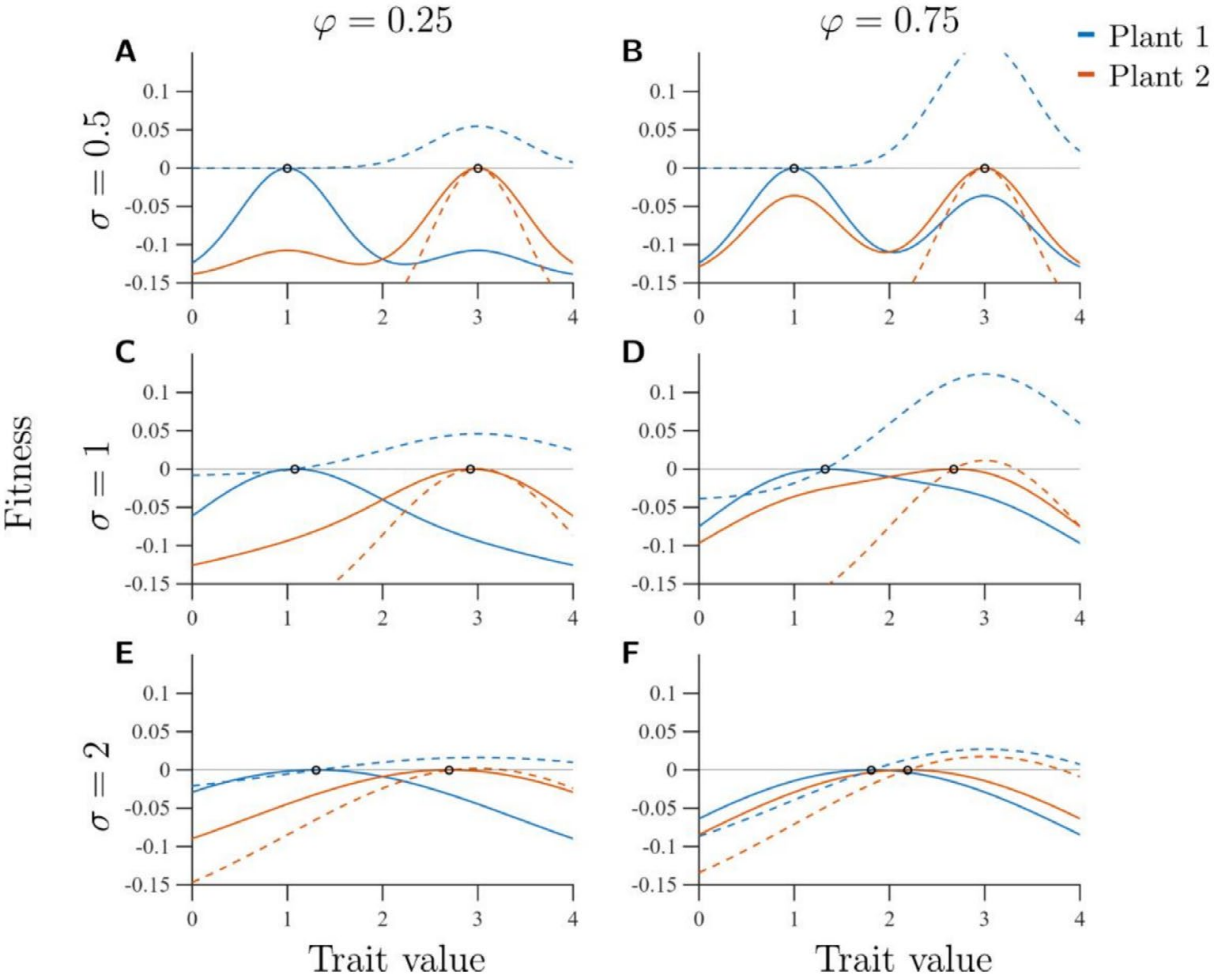
Fitness landscapes at the initial ESS (solid lines; the ESS is indicated by a black circle), and after the abundance of Plant 1 has been changed from 2 to 1 (dashed lines). Blue lines indicate pollinators initially adapted to Plant 1 and red lines indicate pollinators initially adapted to Plant 2. Invasion fitness of 0 is indicated by a horizontal black line. Trait values are shown on the *x*‐axes and invasion fitness values on the *y*‐axes. The left column (A, C, E) shows fitness landscapes for φ=0.25 and right column (B, D, F) fitness landscapes for φ=0.75. The top row (A, B) shows fitness landscapes for σ=0.5, the middle row (C, D) fitness landscapes for σ=1, and the bottom row (E, F) fitness landscapes for σ=2. Remaining parameter values are listed in Table 1. The selection gradient is directed towards the more abundant plant, which in this case is Plant 2 with trait value $\pi_{2}=3$.

When the abundance of a plant changes, selection pressures on the pollinators also change, favouring better matching with the functional trait value of the more abundant plant (Figure 2 and Figure S2). Thus, selection favours functional convergence when the plant composition becomes more homogeneous. Selection gradients are steepest at intermediate values of σ, decreasing gradually as the width of trait matching tolerance increases and more sharply as it decreases (Figure S3). When σ is sufficiently small relative to $\left|\pi_{1}-\pi_{2}\right|$ the selection gradients are so shallow that evolution is negligible (Figure 2A,B). When σ is larger, φ becomes relatively more important than σ: the selection gradient is shallow when φ is small (Figure 2C,E), and steeper when φ is larger (Figure 2D,F).

### 3.3 Eco‐Evolutionary Effects on Pollinator Species Evenness and Functional Diversity

**FIGURE 3:**
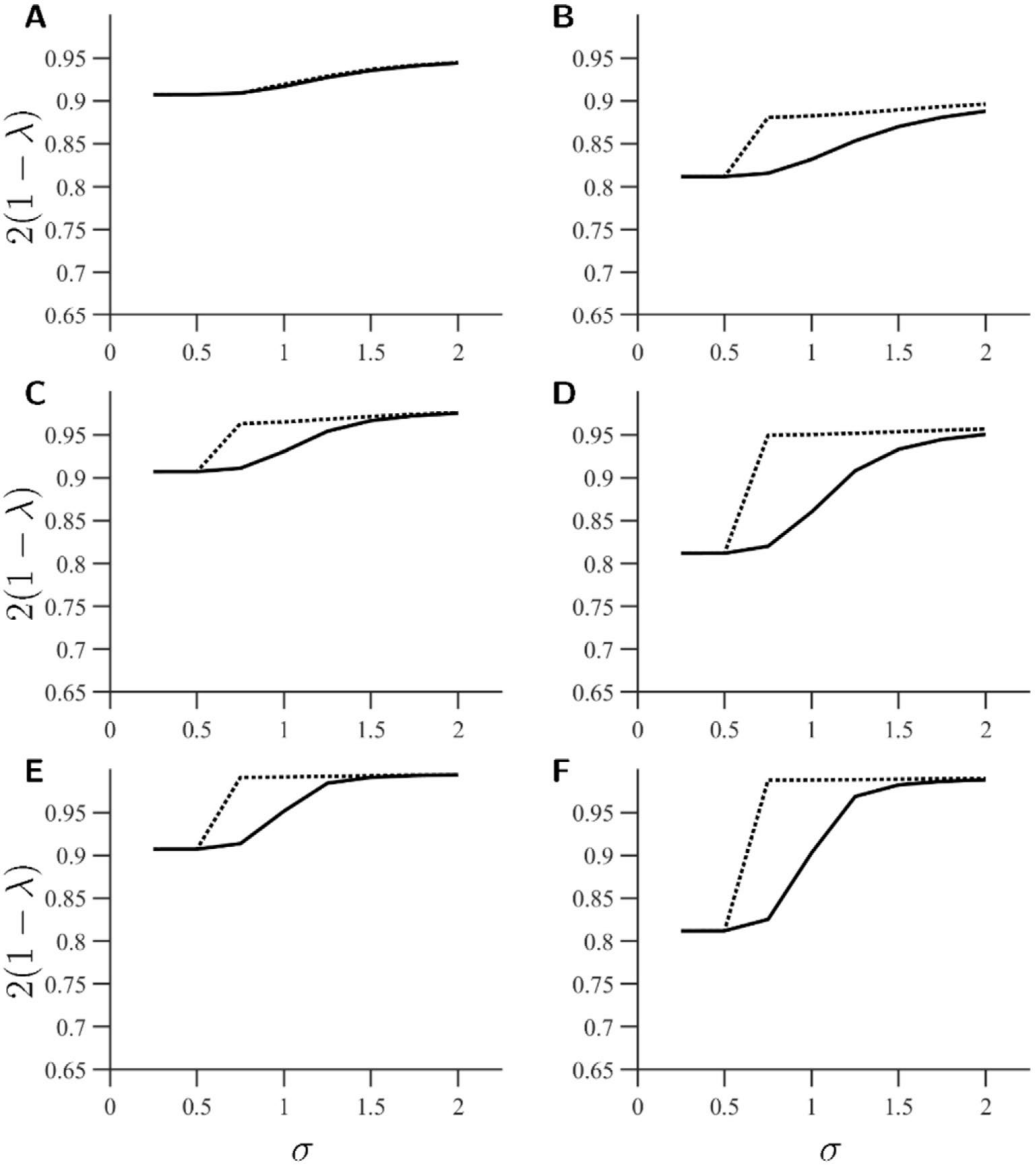
The Gini‐Simpson index reveals that species evenness is eventually restored thanks to evolutionary adaptation of the less well‐adapted species. Solid lines indicate Gini‐Simpson indices at the ecological equilibrium before evolution. Dotted lines indicate Gini‐Simpson indices at the new ESS. Widths of trait matching tolerances (σ) are shown on the *x*‐axis and the Gini‐Simpson indices (scaled to have a maximum of 1) are shown on the *y*‐axis. The left panels (A, C, E) show Gini‐Simpson indices for $K_{1}=3$, and the right panels (B, D, F) show Gini‐Simpson indices for $K_{1}=4$. The upper row (A, B) shows results for φ=0.25, middle row (C, D) for φ=0.5, and lower row (E, F) for φ=0.75. For very narrow trait matching tolerance (σ≤0.5 for $K_{1}=3$ or σ≤0.25 for $K_{1}=4$), no evolution occurs. Remaining parameter values used to create the figure are listed in Table 1.

**FIGURE 4:**
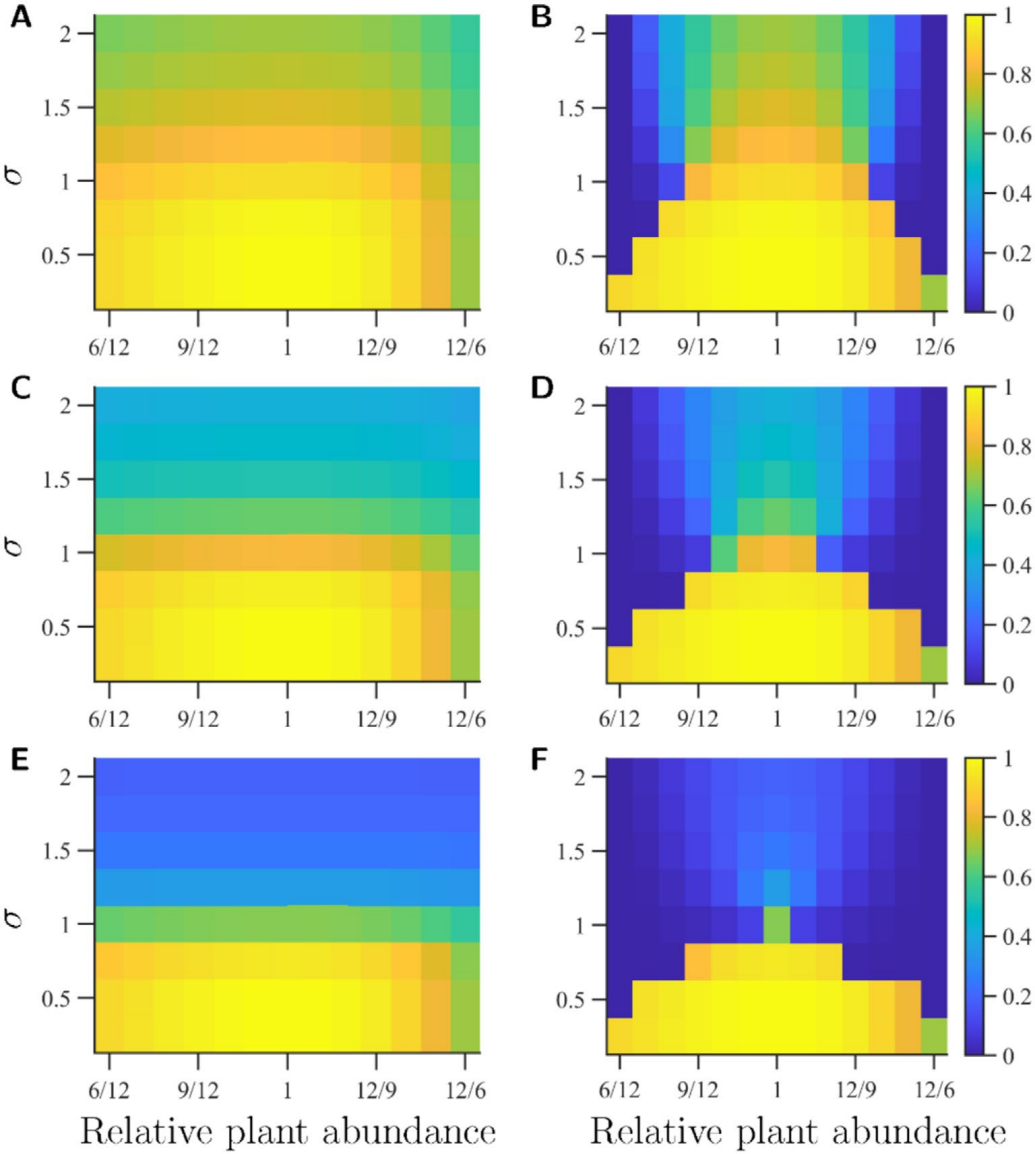
Pollinator functional diversity before and after evolution. Relative plant abundances are shown on the *x*‐axes and widths of trait matching tolerances are shown on the *y*‐axes. The colour bars indicate Rao's quadratic entropy. Results at the ecological equilibrium before evolution are shown in the left panels (A, C, E). Results at the ESS are shown in the right panels (B, D, F). The upper row (A, B) shows results for φ=0.25, middle row (C, D) for φ=0.5, and lower row (E, F) for φ=0.75. Remaining parameter values used to create the figure are listed in Table 1. The functional diversity loss can be considerable even for relatively small asymmetries in plant composition, especially for intermediate σ. The total abundance of plants is increasing from left to right, and consequently the heatmaps are not entirely symmetrical, as the realised species evenness is somewhat higher for lower total abundance of plants.

Before evolution has taken place, the Gini‐Simpson diversity index indicates a considerable decrease in species diversity after a sufficiently large change in plant abundance, especially for small σ (Figure 3, solid lines). After evolution, species evenness is partially restored when σ is large enough (Figure 3, dotted lines). However, as the pollinators adapt to the most abundant plant, the pollinator community becomes less functionally diverse (Figure 4). When the asymmetry in carrying capacities for the plants are large enough, so that one plant dominates the landscape sufficiently, Rao's quadratic entropy can be as low as zero at the ESS, meaning that both pollinator species have identical trait values for their focal pollination traits. Thus, even if evolution may rescue species from changes in plant composition, it may not rescue the functioning of the ecosystem. For intermediate σ and large φ, this can occur although the difference between $K_{1}$ and $K_{2}$ is small.

### 3.4 Long‐Term Effects on Plant Abundance

**FIGURE 5:**
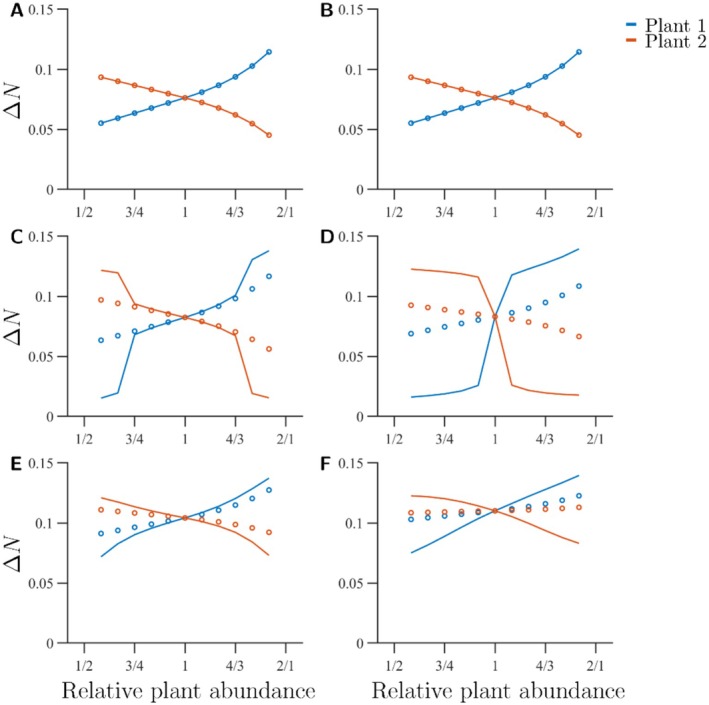
Increase in population growth due to pollination for σ=0.5 (A, B), σ=1.0 (C, D), and σ=2.0 (E, F). The rings represent pollination efficiency before evolution and the solid lines pollination efficiency at the new ESS. Blue lines and rings represent Plant 1, with an abundance that varies from 1 (left) to 4 (right). Red lines and rings represent Plant 2, with a constant abundance of 2. The left panels (A, C, E) show increases in population growth for φ=0.25 and the right panels (B, D, F) show increases in population growth for φ=0.75. Remaining parameter values are listed in Table 1. Larger σ provide more increase in plant population growth than small σ. For sufficiently large σ, the ecological effects are enhanced by evolution. The parameter φ has a large impact on the size of the parameter space where both plants are substantially benefitted by pollination for intermediate σ (cf. panels C and D) but is less important for small or large σ.

The modelled changes in plant abundance are reinforced by eco‐evolutionary feedback loops. The benefit of pollination for the more abundant plant increases as the pollinators evolve towards it, whereas the benefit for the less abundant plant decreases (Figure 5). Again, the effect is particularly strong for intermediate σ and large φ (Figure 5D). When both φ and σ are large, the total abundance of both pollinators increases on an ecological timescale when any plant increases in abundance. This increased pollinator abundance benefits both plants through indirect ecological effects (Figure 5F, dots). After evolution, however, the pollination benefit for the relatively less abundant plant (i.e., Plant 2) becomes reversed and decreases with increased abundance of Plant 1 (Figure 5F, solid lines).

## 4 Discussion

The aim of this study was to use simulations to elucidate how altered plant abundances in agroecosystems influence the eco‐evolutionary dynamics of generalist and specialist pollinators. We found that specialist pollinators exhibit the strongest responses to altered plant composition on ecological timescales. In contrast, pollinators with a moderately generalist trait matching tolerance exhibit the strongest evolutionary responses. Specialist pollinators suffer both strong reductions in abundance and shallow selection gradients as they are unable to utilise the alternative, more abundant resource. Small mutations will thus not noticeably improve the fitness of such specialists, and continuous adaptation is not expected to occur. Super‐generalists also have shallow selection gradients because they can already utilise the alternative, more abundant resource sufficiently and adaptation is thus not required. Moderate generalists can utilise both resources to a limited extent and will gain a significant advantage in fitness by adapting to the abundant alternative resource. Thus, in response to landscape simplification, specialists have a high extinction risk, whereas moderately generalised pollinators undergo evolutionary rescue. This said, our results indicate that although beneficial for the persistence of individual species, evolutionary rescue in landscapes dominated by few plants can be negative for functional diversity of pollinators. When one plant is sufficiently dominant, pollinators undergo convergent evolution (Speed and Arbuckle 2017). In some cases, distinct pollinators evolve ecologically identical functional traits, which in a functional sense would be equivalent to all pollinators but one having gone extinct. Severe losses of functional diversity may thus go unnoticed if one measures only the diversity of species but not their functional properties.

Adaptive loss of functional diversity among pollinators can undermine both ecological integrity and agricultural productivity. Fewer distinct foraging strategies among pollinators can, for example, lead to top‐down effects that disproportionately disadvantage less abundant plant species (Smith et al. 2014; Wei et al. 2021). This, in turn, may accelerate declines in plant diversity, which over time may further weaken pollinator communities. Moreover, loss of functional diversity limits adaptive capacity to future environmental changes (Hoelzel et al. 2019). This is because functionally uniform communities are less likely to contain the genetic and phenotypic variants that tolerate novel or extreme conditions, increasing the risk of pollinator declines and ecological disruption under environmental change. The loss of trait complementarity may also directly reduce ecosystem functioning, including crop pollination, because different functional traits often act synergistically to support stable and efficient pollination services. Because we modelled only one explicit functional trait, yield losses associated with declining functional diversity might therefore in some cases be higher than suggested by our model (Woodcock et al. 2019). Finally, reductions in pollination‐related trait diversity may coincide with functional diversity loss in other traits due to trait correlations (Hederström et al. 2024). This can lead to cascading effects, impacting insect functions beyond pollination, such as resilience to stress, dispersal ability, or interactions with other trophic levels.

Our results have significance for current empirical observations and future empirical tests of theory. On ecological timescales, empirical evidence suggests that pollinators have more overlapping resource preferences when the diversity of plants in the landscape is low (Cappellari et al. 2022) and that generalists often cope better with landscape simplification than specialists (Smith et al. 2014; Weiner et al. 2014), in line with our simulation results. Our results are also consistent with the possibility that specialists under some circumstances may be favoured relative to generalists (Sweeney and Jarzyna 2022). Pollinators that are specialised on the plant that increases in abundance are more strongly favoured by the change than generalists (i.e., the magnitude of both negative and positive numbers in Table 3 are larger for σ=0.5 than for σ=2). Our study thus highlights the importance of a case‐by‐case evaluation of how land‐use change influences pollinators with different strategies for life history and niche. Our theoretical findings are also of significance for empirical results on evolutionary timescales. Even though biodiversity metrics based on species identity, including phylogenetic diversity, are roughly correlated with functional diversity, functional diversity may be low despite high species diversity (Mazel et al. 2018). Our model suggests a partial explanation for this. Convergent evolution of potentially phylogenetically diverse populations, driven by reduced diversity in their mutualistic partners, might result in low functional diversity. Moderate generalists that have had enough time to evolve, might therefore exhibit little correlation between functional diversity and these biodiversity metrics.

Our model has a high level of abstraction, generality, and simplicity. This has the advantage that our results are universal and easy to mechanistically interpret. Thus, our model serves our purpose of illustrating that, in general, evolution may significantly amplify the loss of pollinator function beyond species‐loss. Due to its generality, our model can accommodate any trait relevant for pollination. Different traits would have different optima and different widths of the trait matching tolerance, σ. However, there is necessarily a trade‐off between generality and realism, and we next discuss potential extensions of our model. The model contains only two pollinators and two plants. Other species that may be present in the landscape are treated implicitly. While this allows us to specifically isolate the interactions between the focal species, it overlooks dynamics of more complex plant‐pollinator networks and potential cascades to additional trophic levels. Similarly, fluctuations in the physical environment are not explicitly included in the model. If fluctuations are correlated with flower availability or with the optimum for the focal traits, such as phenology, we expect that stronger fluctuations will drive selection for more generalist strategies. If fluctuations increase over time, for instance if the weather conditions become more unpredictable due to climate change, we expect, based on our results, that adaptation to this may synergistically increase functional diversity loss together with landscape simplification. It would thus be interesting to allow the populations to dynamically adapt to fluctuations in environmental conditions and at the same time accommodate large‐scale effects of eco‐evolutionary cascades to additional species and trophic levels.

We assumed that the plants are domesticated mass‐flowering crops. This means that we assumed that: (1) plants are non‐evolving, as they are not under natural selection, and (2) that flower resources are not limited for pollinators, as they occur in very large amounts. If the plants were allowed to evolve, we expect that evolutionary homogenisation would be further amplified because selection on the less abundant plant would drive adaptation to the more abundant pollinator, and hence towards the more abundant plant. If floral resources were limited, or if evolving plants competed for pollinators, we expect that long‐term coexistence would have been prevented for species whose functional trait values perfectly overlap (Johnson et al. 2022). We would, however, expect some convergent evolution and reduction in functional diversity, in agreement with empirical observations of functional overlap of pollinators in landscapes with low plant diversity, despite strong competition for resources (Cappellari et al. 2022). It would therefore be an interesting extension of our model to include both competition among pollinators for floral resources and evolving plants, as these mechanisms are expected to have opposite effects on pollinator evolution.

## 5 Conclusions

Our trait‐based eco‐evolutionary simulations demonstrate that loss of functional diversity as an adaptive response to landscape simplification can be dramatic even though species diversity is apparently maintained. This loss of pollinator functional diversity may cascade down to plants and thereby reduce agricultural yield of less abundant crops. It may also limit the potential to adapt to future changes, implying possible synergistic effects between biodiversity losses due to land‐use and other forms of environmental change, including climate change. To properly assess the quality of pollination services and their stability, it is thus not enough to focus on the abundance and diversity of species; one also needs to consider their ecological function and phenotypic diversity. Although we had only two species each of pollinators and plants, and one functional trait related to pollination, we expect our qualitative findings to generalise to an arbitrary number of species and to any plant‐pollinator matching trait. Additionally, as our models are general, our findings can be generalised to any mutualistic network, not just plants and pollinators. We conclude that a functional approach to diversity is required to assess how pollination services are influenced by land‐use changes over evolutionary timescales.

## Author Contributions

**Martin Eriksson:** conceptualization (equal), data curation (lead), formal analysis (lead), investigation (lead), methodology (equal), resources (supporting), software (lead), validation (lead), visualization (lead), writing – original draft (lead), writing – review and editing (equal). **Mikael Pontarp:** conceptualization (equal), funding acquisition (lead), methodology (equal), project administration (lead), resources (lead), supervision (lead), visualization (supporting), writing – original draft (supporting), writing – review and editing (equal).

## Conflicts of Interest

The authors declare no conflicts of interest.

## Supporting information


**Figure S1:** Equilibrium population abundances for Pollinator 1 (A) and Pollinator 2 (B) as a function of the carrying capacity of Plant 1. The blue, red, and yellow lines indicate that σ=0.25, σ=1.25, and σ=2.0, respectively. Other parameters: φ=0.5, θ=0.1, $K_{R}=1$.
**Figure S2:** Fitness landscapes for $K_{1}=1$ (A), $K_{1}=1.5$ (B), $K_{1}=3$ (C), and $K_{1}=4$ (D). Other parameters: φ=0.5, σ=1, θ=0.1, $K_{2}=2$, $K_{R}=1$.
**Figure S3:** Magnitude of selection gradient as a function of σ for $K_{1}=1$ and $K_{2}=2$ at the ESS obtained when $K_{1}=K_{2}=2$. Other parameters: φ=0.5, θ=0.1, $K_{R}=1$.

## Data Availability

No data were collected for this study. The code is available in Figshare at https://doi.org/10.6084/m9.figshare.29424932.
