# Supplementary material for: Eco‐Evolutionary Dynamics of Generalist and Specialist Pollinators Facing Plant Diversity Changes
Source: Ecol Evol. 2026 Feb 27;16(3):e73182. doi: 10.1002/ece3.73182 (PMC12949221; doi:10.1002/ece3.73182)
Supplement: Supplementary file 1 — Figure S1: Equilibrium population abundances for Pollinator 1 (A) and Pollinator 2 (B) as a function of the carrying capacity of Plant 1. The blue, red, and yellow lines indicate that σ=0.25, σ=1.25, and σ=2.0, respectively. Other parameters: φ=0.5, θ=0.1, KR=1. Figure S2: Fitness landscapes for K1=1 (A), K1=1.5 (B), K1=3 (C), and K1=4 (D). Other parameters: φ=0.5, σ=1, θ=0.1, K2=2, KR=1. Figure S3: Magnitude of selection gradient as a function of σ for K1=1 and K2=2 at the ESS obtained when K1=K2=2. Other parameters: φ=0.5, θ=0.1, KR=1. [file ECE3-16-e73182-s001.docx]

# Appendix A: Additional figures


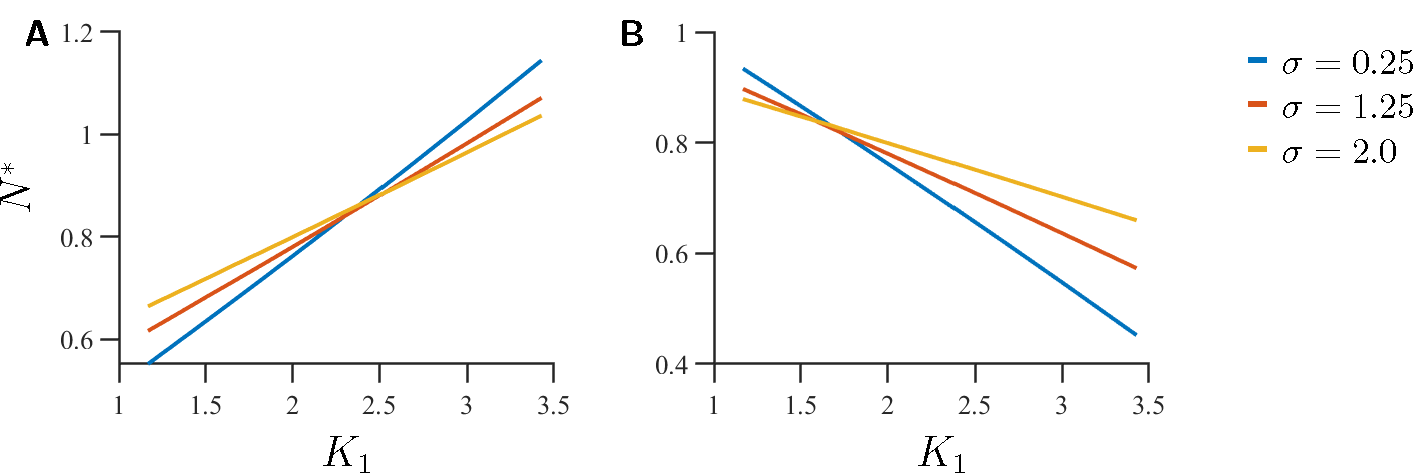


Figure S1: Equilibrium population abundances for Pollinator 1 (A) and Pollinator 2 (B) as a function of the carrying capacity of Plant 1. The blue, red, and yellow lines indicate that $\sigma=0.25$, $\sigma=1.25$, and $\sigma=2.0$, respectively. Other parameters: $\varphi=0.5$, $\theta=0.1$, $K_{R}=1$.


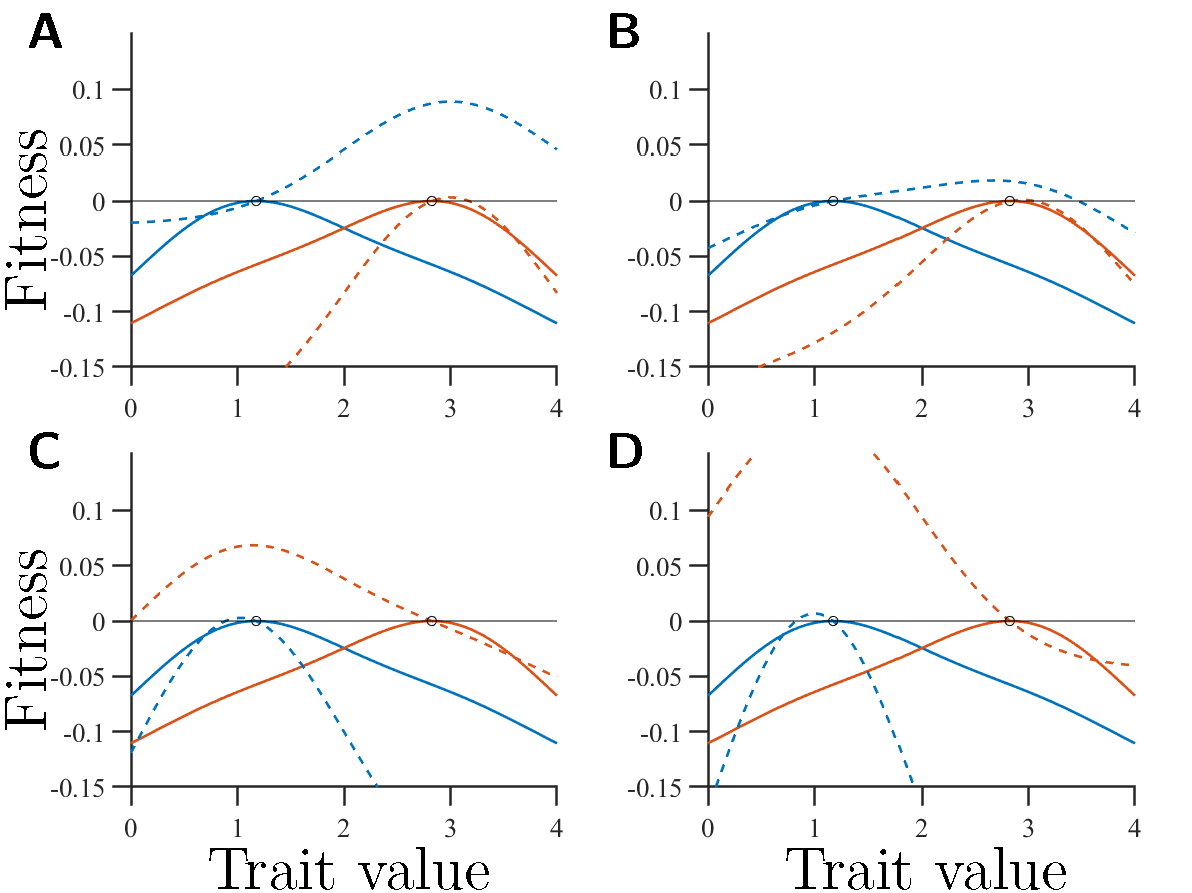


Figure S2: Fitness landscapes for $K_{1}=1$ (A), $K_{1}=1.5$ (B), $K_{1}=3$ (C), and $K_{1}=4$ (D). Other parameters: $\varphi=0.5$, $\sigma=1$, $\theta=0.1$, $K_{2}=2$, $K_{R}=1$.


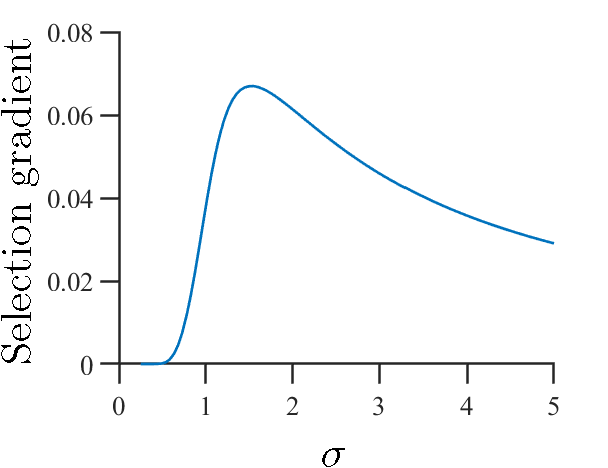


Figure S3: Magnitude of selection gradient as a function of $\sigma$ for $K_{1}=1$ and $K_{2}=2$ at the ESS obtained when $K_{1}=K_{2}=2$. Other parameters: $\varphi=0.5$, $\theta=0.1$, $K_{R}=1$.
